# Pt(iv)-functionalised polyacrylic acid-coated iron oxide magnetic nanoparticles as redox-responsive cancer theranostics†

**DOI:** 10.1039/d5tb01007a

**Published:** 2025-07-01

**Authors:** Beatriz Brito, Thomas W. Price, Cátia V. Rocha, Manuel Bañobre-López, Graeme J. Stasiuk, Juan Gallo

**Affiliations:** a School of Life Sciences, Faculty of Health Sciences, University of Hull Cottingham Road HU6 7RX Hull UK graeme.stasiuk@kcl.ac.uk; b Advanced Magnetic Theranostic Nanostructures Lab, International Iberian Nanotechnology Laboratory Av. Mestre José Veiga 4715-330 Braga Portugal juan.gallo@inl.int; c School of Biomedical Engineering and Imaging Sciences, King's College London St Thomas’ Hospital SE1 7EH London UK

## Abstract

Iron oxide nanoparticles represent a class of nanomaterials with unique physicochemical properties and high potential for theranostic applications. Herein, we functionalised polyacrylic acid (PAA)-coated iron oxide nanoparticles with a chemotherapeutic Pt(iv) prodrug, to prepare Fe_3_O_4_@PAA–Pt(iv) nanostructures that act as *T*_2_ MR theranostics with redox- (and thus TME-) responsive therapeutic properties. The synthesis of Fe_3_O_4_@PAA–Pt(iv) nanoparticles was optimised to yield nanoparticles with appropriate hydrodynamic diameter and Pt/Fe ratio. The Fe_3_O_4_@PAA–Pt(iv) nanoparticles displayed promising magnetic and relaxometric properties, showing a higher relaxivity than commercially available NP-based MRI agent Resovist®. Cell internalisation studies in 2D and 3D cell models demonstrated that the nanomaterials accumulated in cancer cells after only 6 h of incubation at a concentration that allowed for contrast enhancement in MRI. Cell viability studies showed that Fe_3_O_4_@PAA–Pt(iv) nanoparticles were 2.5 times more effective than the Pt(iv) prodrug in inducing apoptosis (IC_50_ = 156 μM *vs.* 379 μM) in 2D models, while in 3D models, they were found to be as effective as active drug cisplatin. These results show the potential of these versatile Pt(iv)-functionalised PAA-coated iron oxide nanostructures as redox responsive MR theranostics for cancer therapy.

## Abstract 2

A novel redox-responsive theranostic nanocomposite was developed to deliver a platinum prodrug to solid tumors.

## Graphical abstract

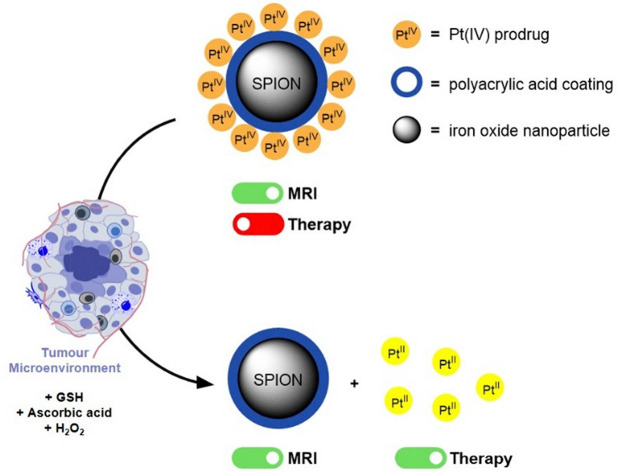

## Introduction

Theranostics represent a turning point in cancer therapy by combining medical imaging and therapeutic functionalities into a single platform, to enable real-time monitoring of drug delivery, therapeutic response, and disease progression.^1–3^ Smart or responsive theranostics go a step further, by integrating therapeutic and/or imaging components capable of undergoing structural or physicochemical alterations to activate their functions in response to an exogenous (*e.g.* light) or endogenous (*e.g.* redox environment) signal upon reaching the target tissue.^1,4–6^ Regarding endogenous triggers, responsive theranostic agents have been designed to be sensitive to a reducing environment,^4^ enabling the activation of their therapeutic and/or imaging functions in redox altered environments. A direct application of these theranostics comes in oncology where a marked difference is found between diseased and healthy tissues. In the tumour microenvironment (TME) or inside cancer cells, glutathione (among other redox active species) is overexpressed, with concentrations in the TME up to four times higher than in healthy tissues.^7–10^

Among the various materials explored for theranostic applications, superparamagnetic iron oxide nanoparticles (SPIONs) have emerged as promising candidates due to their unique physicochemical properties, including superparamagnetism, enhanced magnetic susceptibility, biocompatibility, and ease of surface functionalisation.^11–13^ Given these properties, SPIONs have been widely investigated as drug delivery carriers, magnetic hyperthermia (MH) effectors and magnetic resonance imaging (MRI) contrast agents.^14^

MRI is an imaging modality that provides highly detailed three-dimensional images of organs and tissues in the body and is used to detect a wide variety of pathological conditions, including cancer.^15^ MRI works by using strong magnetic fields and radio waves to visualise water proton nuclei in the body. Due to the high abundance of water molecules in biological systems, the signal to noise ratio of MRI is low. MRI contrast agents (CAs), including SPIONs, are widely used to increase the sensitivity of MR and thus assist in the diagnosis of several pathologies.^16–19^ SPIONs are commonly known as *T*_2_ contrast agents, as they shorten the *T*_2_ relaxation time, enhancing contrast in *T*_2_-weighted images. While gadolinium-based *T*_1_ contrast agents are more widely used in the clinic, SPIONs offer significant advantages as contrast agents, including greater biocompatibility and biodegradability and higher relaxivity values.^20^

Regarding the therapeutic component, platinum (Pt)-based chemotherapeutics, particularly cisplatin and its derivatives, have been extensively employed in cancer treatment in the clinic, particularly in the treatment of lung and ovarian carcinomas.^21,22^ Cisplatin is a Pt(ii)-based chemotherapeutic drug that induces apoptosis in cancer cells by binding to DNA and inducing intra-strand cross-linking.^22^ However, the use of cisplatin and its derivatives is often limited by severe side effects and drug resistance mechanisms.^21,22^ In order to circumvent some of these limitations, Pt(iv) prodrugs have been explored as promising alternatives to reduce off-target effects. Pt(iv) complexes can work as redox-responsive prodrugs that are reduced on site to the active Pt(ii) species in the presence of reductive environments, such as those of TME and cancer tissues.^23–26^ The redox-triggered activation of Pt(iv) prodrugs to active Pt(ii) drugs allows the targeted action of activated drug in cancerous tissues, thus mitigating systemic toxicity and improving therapeutic efficacy. Furthermore, the addition of axial ligands on Pt(iv) complexes can impact the overall properties of the prodrugs, such as lipophilicity or redox potentials.^27,28^ Lipophilicity can then have an effect on cellular entry pathways, with some Pt(iv) complexes being preferentially up taken by passive diffusion and others by transporter-mediated transport.^29^ The redox potential of the Pt(iv) complexes can influence reduction processes of Pt(iv) complexes, leading to quicker or slower reduction.^27^

As such, functionalising SPIONs with Pt(iv) prodrugs would allow for passively targeted drug delivery to tumours, due to the enhanced permeability and retention (EPR) effect,^30–32^ and controlled release of active drug in the reductive TME.

**Fig. 1:**
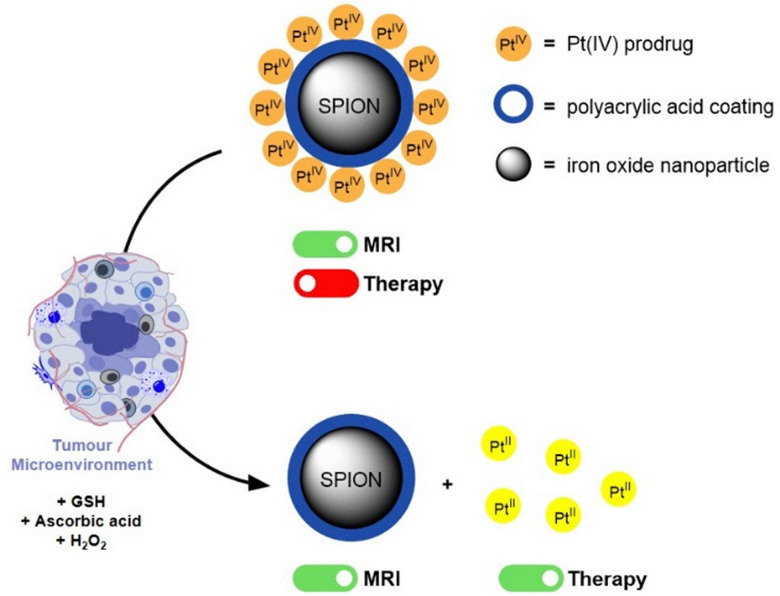
Schematic representation of the mechanism through which Fe_3_O_4_@PAA–Pt(iv) nanoparticles induce activation of apoptotic pathway in response to biologically available reducing agents in tumour microenvironments.

While several studies have described advances in cancer nanotheranostics^4,33–35^ and a few have even described systems combining iron oxide nanoparticles with Pt(iv) prodrugs,^36–38^ further work is still required to ensure optimal imaging and therapeutic functionalities, as well as biocompatibility. As such, in this work, we describe a simple preparation of poly(acrylic acid) (PAA)-coated and Pt(iv)-functionalized iron oxide structures: Fe_3_O_4_@PAA–Pt(iv) nanoparticles. In this system, PAA was employed as a versatile coating material that not only enhances colloidal stability and biocompatibility, but also facilitates surface functionalisation.^39^ Being able to control the ratio of chemotherapeutic effector to MR imaging agent (in this case Pt/Fe) is essential in this system, as the acquisition of diagnostically relevant MR images requires a higher concentration of contrast agent than the concentration of chemotherapeutic drug necessary to induce cytotoxicity in pathological tissues. As such, having a PAA coating on the surface of the iron oxide nanoparticles with a high density of reactive groups, allows for the preparation of a highly adaptable system with controllable MR and therapeutic functionalities. Furthermore, these Fe_3_O_4_@PAA–Pt(iv) nanoparticles provide environmentally (redox and tumour microenvironment) switchable (off/on) therapy, meaning that the Pt(iv) prodrugs on the surface of the nanoparticles are activated to Pt(ii) active drug cisplatin only in response to reducing agents commonly found in the TME (Fig. 1). By integrating redox-responsive Pt(iv) chemotherapy with MRI contrast enhancement, this system provides a multifaceted platform for improved cancer diagnosis and treatment. Indeed, these theranostics could even prove useful in the treatment of several types of cisplatin-resistant cancers, by circumventing inactivation resistance mechanisms usually dependent on reducing agents in TME and cancer cells.^4^

## Results and discussion

### Synthesis and characterisation of Fe_3_O_4_@PAA nanoparticles

Polyacrylic acid-coated iron oxide magnetic nanoparticles (Fe_3_O_4_@PAA nanoparticles) were synthesised in aqueous medium following a modified hydrothermal protocol (ESI,† Scheme S1). PAA was used as a stabilising agent to prevent aggregation of the nanoparticles, as well as to provide opportunities for facile nanoparticle functionalisation, given the high density of reactive functional groups (carboxylic acids) in PAA.^39^

**Fig. 2:**
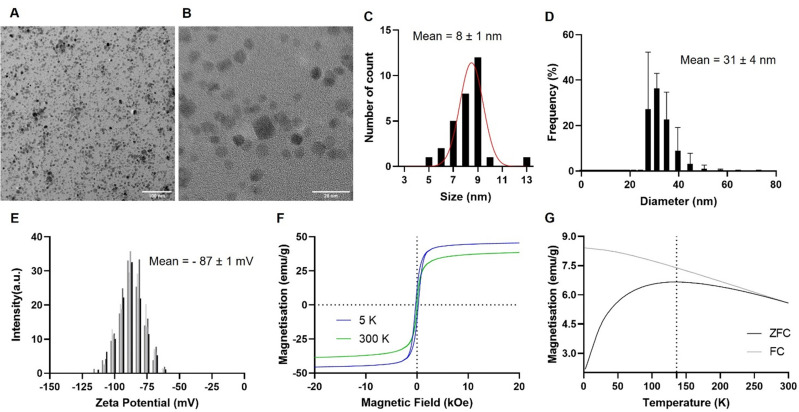
Representative bright-field TEM images of Fe_3_O_4_@PAA NPs, with scale bar representing (A) 100 nm and (B) 20 nm. (C) Size distribution of nanoparticles’ metal core, as measured by TEM. (D) Size distribution of nanoparticles’ hydrodynamic size, as measured by DLS, in H_2_O (pH = 7.4). (E) Zeta potential measurements for Fe_3_O_4_@PAA NPs in H_2_O (pH = 7.4). Hysteresis loop of magnetization (in emu g^−1^) at applied magnetic fields ranging from −20 to 20 kOe, as measured by (F) SQUID at 5 K (blue) and 300 K (green) and (G) ZFC–FC measurements of Fe_3_O_4_@PAA nanoparticles at 100 Oe.

Morphological analysis of these nanoparticles was carried out using transmission electron microscopy (TEM, Fig. 2(A) and (B)), which showed the synthesised nanoparticles as well-defined crystalline pseudo-spheres with an average inorganic core diameter of 8 ± 1 nm (Fig. 2(C)). Dynamic light scattering (DLS) was used to determine the hydrodynamic size (*D*_h_) and the surface charge (*ζ*-pot) of these nanomaterials. Results indicate that the hydrodynamic size was larger than the core size (31 ± 4 nm, Fig. 2(D)), as expected, due to the presence of the PAA polymer on the surface of the nanoparticles. The zeta potential of these systems in water (pH = 7) was highly negative (−87 ± 1 mV, Fig. 2(E)), also as expected,^40^ since the polymer presents negatively charged carboxylic acid groups on the surface of the nanoparticles.

Powder X-ray diffraction (XRD) was then used to confirm that the iron-based core of the nanoparticles was made of magnetite crystals (ESI,† Fig. S1A). Inductively coupled plasma optical emission spectroscopy (ICP-OES) allowed for the determination of the iron content in these nanosystems: 284.7 mM, and TGA studies allowed for the determination of the ratio between the mass of polymer and inorganic matter in the nanoparticles (*n*_organic_/*n*_inorganic_ = 24.6, ESI,† Fig. S1B). The optical absorption spectrum of the magnetite nanoparticles revealed an absorption maxima of iron oxides between 350 and 400 nm (ESI,† Fig. S1D), as expected for nanoparticles of this size.^41–43^ Fourier transform infrared spectroscopy (FTIR) was then used to confirm the presence of the PAA polymer on the surface of the nanostructures (ESI,† Fig. S1E). The obtained spectra for the Fe_3_O_4_@PAA NPs shares similarities with the spectra of the polymer precursor, such as peaks at 1396, 1554 and 1662 cm^−1^, corresponding to CH_2_ bending (*δ*(CH_2_)), asymmetric –COO^−^ stretching (*ν*(COO^−^)) and *ν*(C=O), respectively.^44^ Overall, these results confirmed that Fe_3_O_4_@PAA NPs were composed of magnetite cores and had a PAA coating, which was relevant to the following functionalisation steps.

The magnetic properties of the Fe_3_O_4_@PAA NPs were then investigated by superconducting quantum interference device (SQUID) as performance of the particles as MR contrast agents is heavily associated to their magnetic properties.^30^ The magnetic hysteresis loops determined by SQUID (Fig. 2(F)) are typical of superparamagnetic materials, as the nanoparticles could be easily magnetised and exhibited a quick equilibration magnetisation and relatively high magnetisation saturation (*M*_s_) values (38 emu g^−1^ measured, at 300 K).^45–48^ The magnetization curves of the iron oxide nanoparticles measured at 300 K also demonstrate a superparamagnetic behaviour by showing almost no remanence (*M*_r_ ≤ 2 emu g^−1^) and coercivity (*H*_c_ ≤ 0.03 kOe). To further clarify the origin of these magnetic properties, SQUID measurements were performed at 5 K. At this temperature, the magnetisation saturation, the remanence and the coercivity values increased when compared to results at 300 K (*M*_s_ increased from 38 emu g^−1^ to 46 emu g^−1^, *M*_r_ increased from 2 emu g^−1^ to 12 emu g^−1^ and *H*_c_ went from 0.03 kOe to 0.26 kOe, at 300 K and 5 K, respectively). This is a strong indication of a typical superparamagnetic behaviour, where below a certain temperature (blocking temperature) a magnetically blocked state exists. This was further confirmed by measuring the zero-field-cooled, field-cooled (ZFC–FC) magnetisation (Fig. 2(G)), indicating that the transition from superparamagnetic to the magnetically-blocked state occurs at ∼136 K. Altogether, these results confirm that Fe_3_O_4_@PAA nanoparticles behave as superparamagnetic nanoparticles within the application temperature range.

### Synthesis and characterisation of Fe_3_O_4_@PAA–Pt(iv) nanoparticles

To produce theranostics agents, we coupled Fe_3_O_4_@PAA nanoparticles with Pt(iv) complex dihydroxycisplatin (DHC, ESI,† Scheme S1 and Fig. S2) by formation of an ester bond between the free carboxylic groups on the surface of nanoparticles 1 and the hydroxyl groups on the Pt(iv) complex, using peptide coupling reagents. There were two main outcomes of this reaction that had to be optimised: the Pt/Fe ratio and the hydrodynamic size of the final Fe_3_O_4_@PAA–Pt(iv) nanoparticles.

It is important to note that, as with all theranostics, but in particular with MR theranostics, the ratio of therapeutic agent to imaging agent must be carefully controlled, to ensure optimal efficiency of both imaging and therapeutic functions.^1^ The dose of intravenous Fe (in iron oxide nanoparticles) administered to mice for MRI is usually in the range of 2 to 20 mg kg^−1^ (35.8–358 μmol kg^−1^),^49–52^ while a single dose of intravenous cisplatin administered to mice for chemotherapy is 5–6 mg kg^−1^ (17–20 μmol kg^−1^).^53^ As such, to make promising dual functional agents, the optimal molar ratio between the Pt dose for chemotherapy and the Fe dose for MR (Pt/Fe) is between 0.05 to 0.5.

Additionally, since the Pt(iv) complex herein used has 2 hydroxyl groups available, specific reaction conditions might favour the bridging of two nanoparticles through a single Pt(iv) complex, which will induce the formation of nanoparticle aggregates. As such, having a close control of the reacting Pt(iv) to nanoparticle ratio allows an easier optimisation of the system.

The molar concentration of Fe_3_O_4_@PAA nanoparticles (0.024 mM) and their molecular weight (1 686 880 g mol^−1^) were estimated by combining data acquired from TEM, ICP and TGA experiments. The optimal molar ratio of Pt/Fe in the theranostic probes of 0.05 to 0.5, corresponds to around 600 to 6000 Pt(iv) complexes per Fe_3_O_4_@PAA nanoparticle.

Preliminary feasibility studies were performed to determine whether sufficient carboxylic acids on the surface of nanoparticles 1 were available to achieve the predicted optimal ratio. These tests were performed by combining different amounts of the nanoparticles with a constant amount of fluorescein cadaverine (ESI,† Scheme S2) in the presence of peptide coupling reagents. This gave a number of 9.42 × 10^−4^ mmol of available carboxylic acids (ESI,† Fig. S3E), corresponding to around 800 carboxylic acids on the surface of the nanoparticles available for functionalization reactions. As the Pt(iv) complex is significantly smaller than fluorescein cadaverine, even more carboxylic groups on the nanoparticles were expected to react with the prodrugs, due to reduced steric hindrance.

A systemic optimisation study was then conducted to determine the optimal reaction conditions for the synthesis of Fe_3_O_4_@PAA–Pt(iv) nanoparticles. First, the effect of the amount of Pt(iv) complexes added during synthesis on nanoparticle aggregation (ESI,† Table S1) was investigated. These studies showed that at high Pt(iv)/NPs ratio, the hydrodynamic size of the nanoparticles increases and aggregates are formed, inferring that a bidentate configuration is produced in these cases.

Following this study, the concentration of Fe_3_O_4_@PAA nanoparticles on the formation of aggregates was investigated (ESI,† Table S2). DLS results showed that more diluted reaction mixtures (*V*_reaction_ = 100 × *V*_NPs_) yielded nanoparticles of around 55 nm, while more concentrated conditions (*V*_reaction_ = 10 × *V*_NPs_) led to the production of nanoparticle aggregates of 766 nm. As such, to avoid the formation of aggregates, the synthesis of Fe_3_O_4_@PAA–Pt(iv) NPs was carried out at *V*_reaction_ = 100 × *V*_NPs_. Next, the Pt/Fe ratio in the Fe_3_O_4_@PAA–Pt(iv) nanoparticles was optimised by varying the amount of nanoparticles 1 while keeping the Pt(iv) complex and coupling reagents concentrations unchanged (ESI,† Table S3). ICP measurements for the synthesised nanoparticles showed that conditions D to G (ESI,† Table S3) allowed for the preparation of Fe_3_O_4_@PAA–Pt(iv) nanoparticles with Pt/Fe within the optimal range of 0.05 to 0.5. Fe_3_O_4_@PAA–Pt(iv) nanoparticles were then prepared in bulk using similar conditions to F and G, since these allowed for the synthesis of nanoparticles with the highest Pt/Fe ratios within the described range.

**Fig. 3:**
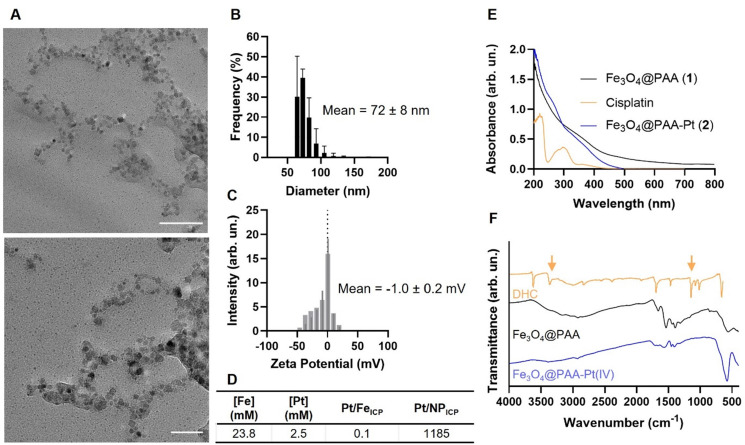
(A) Representative bright-field TEM images of Fe_3_O_4_@PAA–Pt(iv) NPs, with scale bar representing 100 nm (above) and 50 nm (below). (B) Size distribution of nanoparticles’ hydrodynamic size, as measured by DLS, in H_2_O (pH = 7.4) and (C) zeta potential measurements. (D) Table summarising ICP results. (E) UV-Vis spectra of NPs 1, 2 and cisplatin, in H_2_O (pH = 7.4). (F) FTIR spectra of NPs 1, 2 and Pt(iv) complex (DHC).

TEM images of the Fe_3_O_4_@PAA–Pt(iv) nanoparticles showed that the addition of the Pt(iv) complex on the nanoparticles did not significantly alter the morphology of the nanoparticles (Fig. 3(A)). The hydrodynamic size of the nanoparticles increased from 31 nm to 72 nm (Fig. 3(B)), remaining within the target size range for biological applications.^54,55^ The zeta potential of these systems in water (pH = 7) was negative but very close to neutral (−1.0 mV ± 0.2 mV, Fig. 3(C)), due to the neutralization of the carboxylate groups on the surface of the NPs by the Pt(iv) complexes. The Pt(iv) functionalization was also confirmed by ICP-OES, which indicated a Pt/Fe ratio of 0.11, equivalent to around 1185 Pt complexes per nanoparticle (Fig. 3(D)), which is within the range that would simultaneously allow for optimal imaging and therapeutic functions. Finally, DLS was also used to investigate the long-term stability of the final probes. The hydrodynamic size measured two years after preparation was 49.6 ± 5 nm, not too dissimilar to the original size.

The absorbance spectrum for NPs 2 (Fig. 3(E)) showed an absorption shoulder at 260 nm, which was attributed to the surface platinum groups. Comparison of the FTIR spectra of NPs 1 and the Pt(iv) complex (Fig. 3(F)) supports the binding of the axial ligands of the complex to the nanoparticles, since the peaks at 3510 and 1040 cm^−1^, which correspond to the *ν*(Pt–OH) and the *δ*(PtO–H), were not present in the spectrum of NPs 2.^56^ These results might suggest that the complexes are binding more than one carboxyl group at the same time, either in the same nanoparticle or by bridging two nanoparticles. The former scenario is more likely, as DLS for these materials does not show clear evidence of aggregate formation. Still, peaks at 3250, 1580 and 552 cm^−1^, corresponding to *ν*(NH_3_), *δ*(NH_3_) and *ν*(PtO) respectively, appear on both spectra, indicating the presence of Pt(iv) complexes on the surface of the magnetite particles.

XPS was then used to study the oxidation state of Pt in the nanoparticles (ESI,† Fig. S5).^4,57^ In the case of our nanostructures, three peaks can be observed at 73.0 eV, 76.1 eV and 78.9 eV. A curve-fitting procedure was applied to discriminate all the peak components, revealing two doublets: one attributed to Pt(ii) (Pt4f_7/2_ = 73.0 eV and Pt4f_5/2_ = 76.2 eV), while the other doublet attributed to Pt(iv) (Pt4f_7/2_ = 75.7 eV and Pt4f_5/2_ = 78.9 eV).^58^ This indicates that both Pt(ii) and Pt(iv) are present in the prepared nanosystems, with approximately 32% of the Pt in the system being in the +4 oxidation state. Further optimisation studies should focus on the preparation of nanoparticles exclusively made of Pt(iv).

### Relaxometric properties of Fe_3_O_4_@PAA and Fe_3_O_4_@PAA–Pt(iv) NPs

The efficacy of Fe_3_O_4_@PAA and Fe_3_O_4_@PAA–Pt(iv) nanoparticles as *T*_2_ MR contrast agents was then evaluated in relaxometry and MRI studies at two different clinical fields.

Relaxometric studies at 1.5 T established that Fe_3_O_4_@PAA nanoparticles had *r*_2_ = 141.6 mM^−1^ s^−1^ (ESI,† Fig. S4B), which is higher than the relaxivity of commercial, iron oxide-based CA Resovist® (98.4 mM^−1^ s^−1^ at 1.5 T, *D*_H_ = 60 nm).^59^ The Pt(iv)-functionalised nanoparticles had *r*_2_ = 215.6 mM^−1^ s^−1^ (ESI,† Fig. S4D) and a higher *r*_2_/*r*_1_ ratio when compared to NPs 1 (*r*_2_/*r*_1_ = 28 *versus* 7.5 for NPs 2 and 1, respectively). It has been reported that coating chemistry and surface functional groups can significantly alter the relaxivity of iron oxide nanoparticles^60,61^ by having an effect on the chemical exchange and diffusion of protons in the coating layer and by influencing suspension stability, respectively. Given that the functionalised nanoparticles present a less negative charge when compared to the Fe_3_O_4_@PAA NPs (−1.0 ± 0.2 *versus* −87 ± 1 mV for NPs 2 and 1, respectively), they are indeed expected to have a higher *r*_2_/*r*_1_ ratio^60,62^ and thus a better performance as *T*_2_ contrast agents.

**Fig. 4:**
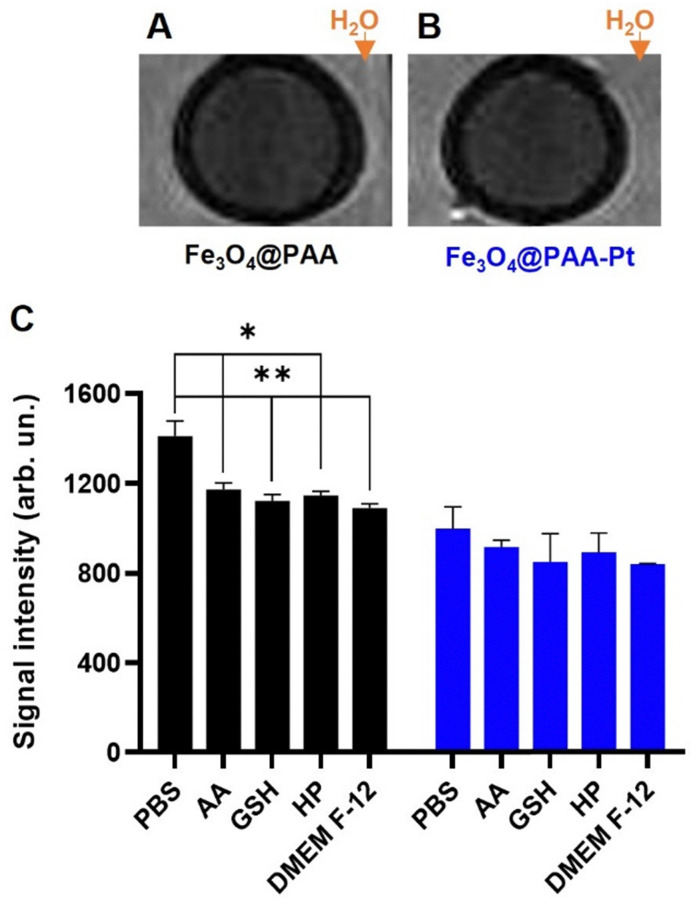
*T*_2_-weighted phantom image of (A) Fe_3_O_4_@PAA and (B) Fe_3_O_4_@PAA–Pt(iv) nanoparticles (455 μM of Fe) in PBS, surrounded by water, at 3.0 T. (C) *T*_2_-weighted MR signals of Fe_3_O_4_@PAA (black, 200 μM of Fe) and Fe_3_O_4_@PAA–Pt(iv) nanoparticles (blue, 200 μM of Fe) in the presence of 100 μM of ascorbic acid (AA), glutathione (GSH), hydrogen peroxide (HP), and DMEM F-12 medium, at 3.0 T, *p < 0.035, **p < 0.02. Error bars indicate standard deviation (SD).

To confirm that these nanoparticles could be used as *T*_2_ MR CAs, *T*_2_-weighted MR phantom images of NPs 1 and 2 (455 μM of Fe) were acquired using an MR scanner working at a clinical field of 3.0 T (Fig. 4(A) and (B)). The acquired images showed considerable contrast generation from both the Fe_3_O_4_@PAA and Fe_3_O_4_@PAA–Pt(iv) nanoparticles, when compared to the surrounding water. *T*_2_-Weighted images of the Fe_3_O_4_@PAA and Fe_3_O_4_@PAA–Pt(iv) nanoparticles were also taken in PBS in the presence of different reducing agents (ascorbic acid – AA, glutathione – GSH, hydrogen peroxide – HP), and in a suspension in DMEM-F12 media, to evaluate the effect of these conditions on the MR properties of the nanoparticles (Fig. 4(C)). Results show that the *T*_2_ signal from Fe_3_O_4_@PAA–Pt(iv) nanoparticles was consistent throughout these conditions, while the contrast generated by Fe_3_O_4_@PAA nanoparticles varied only slightly upon addition of reducing agents or cell media. Overall, these results confirm that Fe_3_O_4_@PAA–Pt(iv) nanoparticles have promising imaging capabilities.

### *In vitro* theranostic evaluation of Fe_3_O_4_@PAA–Pt(iv) nanoparticles

To further evaluate the effectiveness of these nanostructures as MR contrast agents and therapeutic agents, *in vitro* studies were performed in A549 human non-small-cell lung carcinoma cells. This cell line was chosen because cisplatin has been used in the treatment of non-small-cell lung carcinoma since the late 1970s.^63^

**Fig. 5:**
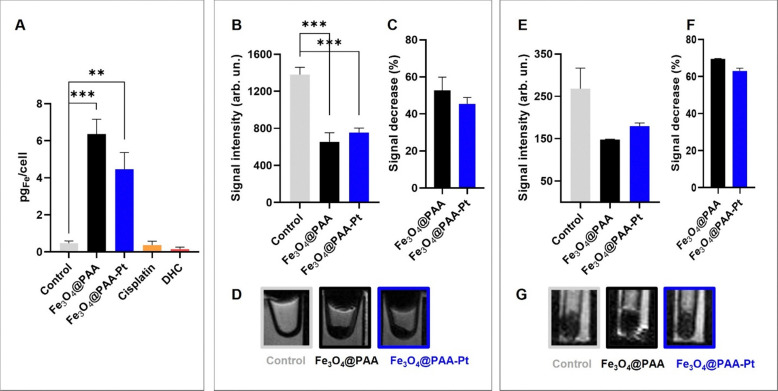
Internalisation of iron oxide NPs 1 and 2 after 6 h of treatment at [Fe] = 500 μM and [Pt] = 50 μM (when appropriate). (A) Internalisation study performed with 2D cell models of A549 cells, using ICP to determine the concentration of internalised Fe (*n* = 2), **p = 0.0015, ***p < 0.0001 (one-way ANOVA test). Internalisation of 2D cell models of A549 cells observed using *T*_2_-weighted MR images of cell pellets (D) and corresponding *T*_2_ signal enhancement (B, one-way ANOVA test, ***p < 0.0006) and % signal decrease (C, *n* = 2), at 3 T. Internalisation of 3D cell models of A549 cells observed using *T*_2_-weighted MR images of cell spheroids (G) and corresponding *T*_2_ signal enhancement (E) and % signal decrease (F, *n* = 2), at 3 T. Error bars indicate standard error of mean (SEM).

First, the efficacy of the nanoparticles as *T*_2_ contrast agents was evaluated by investigating their ability to accumulate in 2D and 3D A549 cells cultures and induce a *T*_2_ signal decrease in MRI. Internalisation studies in 2D cell models were employed to qualitatively and quantitatively evaluate the cellular uptake of Fe_3_O_4_@PAA and Fe_3_O_4_@PAA–Pt(iv) after 6 h of incubation, by MRI and ICP. Briefly, A549 cells (2 × 10^5^ cells per well) were seeded and incubated for 24 h. The cells were then treated with the different compounds ([Fe] = 500 μM, [Pt] = 50 μM) and incubated for 6 h, after which cells were thoroughly washed with PBS, trypsinised and either treated with acid for ICP evaluation or pelleted and imaged in the MR scanner. ICP experiments (Fig. 5(A)) detected a statistically significant increase in the intracellular iron content of 2D cells treated with both Fe_3_O_4_@PAA and Fe_3_O_4_@PAA–Pt(iv) NPs compared to the control conditions, which infers that the nanoparticles were successfully internalised. Evaluation of the *T*_2_-weighted images (Fig. 5(D)) showed a statistically significant decrease in the signal intensity in iron oxide nanoparticle-treated *versus* untreated cells (Fig. 5(B)). Since nanoparticles 1 and 2 act as *T*_2_, or darkening contrast agents, the observed signal decrease confirms the nanoparticles were internalised by the cells after 6 h (Fig. 5(C)). No differences were observed in the internalization of NPs 1 and 2. Since 3D cell models are more complex systems better able to recapitulate the *in vivo* tumour microenvironment,^64–67^ the MR internalisation study was repeated in 3D cell models of the same A549 cell line. Briefly, A549 3D cells were grown in faCellitate BIOFLOAT™ plate for 3 days; spheroids were then collected and treated with nanoparticles 1 and 2 ([Fe] = 500 μM) for 6 h (4 spheroids per well), washed, placed inside capillary tubes and imaged. Evaluation of the *T*_2_-weighted images (Fig. 5(G)) showed a clear decrease in the signal intensity of spheroids treated with 1 or 2*versus* untreated spheroids (Fig. 5(E) and (F)), as expected. This data might also indicate that nanoparticle internalisation takes longer in the more complex 3D systems. Nonetheless, these results indicate that Fe_3_O_4_@PAA–Pt(iv) NPs are internalised by A549 cells, to provide a significant MR contrast, suggesting that these nanostructures could be used for MR imaging purposes.

**Fig. 6:**
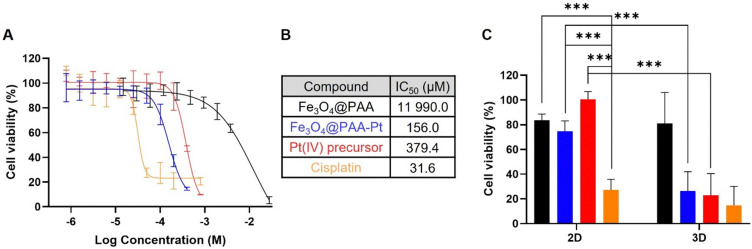
Cell viability studies to test toxicity of Fe_3_O_4_@PAA NPs (1, black), Fe_3_O_4_@PAA–Pt(iv) NPs (2, blue), Pt(iv) precursor (red), and cisplatin (yellow). (A) Cell viability study employing 2D models of A549 cells after 48 h of treatment and corresponding (B) calculated IC_50_ values (*n* = 3) for the different compounds. Concentration refers to [Pt] for all compounds except Fe_3_O_4_@PAA NPs, in which it refers to [Fe]. (C) Comparison of cell viability in 2D and 3D A549 cell cultures after 48 h of treatment with cisplatin, Pt(iv) precursor and NPs 1 and 2, at a concentration of 100 μM of Pt (*n* = 2), ***p < 0.0001 (two-way ANOVA analysis on GraphPad Prism). Error bars indicate SD.

To evaluate the effectiveness of these nanostructures as therapeutic effectors, *in vitro* toxicity studies were performed using the same cell line. The cytotoxic effect of the Fe_3_O_4_@PAA–Pt(iv) nanoparticles in A549 2D cell models was first compared to that of the active drug cisplatin, the Pt(iv) precursor prodrug (DHC), and Fe_3_O_4_@PAA nanoparticles (Fig. 6(A)).^68^ Importantly, Fe_3_O_4_@PAA nanoparticles showed good biocompatibility, as they presented an IC_50_ of 12.0 mM (Fig. 6(B)). The toxicity of the nanosystem 2 in the 2D model (IC_50_ = 156.0 μM) was considerably higher than that of the precursor Pt(iv) prodrug (IC_50_ = 379.4 μM), either due to a synergistic effect between Fe and Pt or due to the enhanced delivery of the Pt(iv) prodrug in nanoparticle form (and thus higher intracellular concentrations of the prodrug are reached). Nonetheless, these 2D cell viability studies showed a clear cytotoxicity difference between the active drug cisplatin (IC_50_ = 31.6 μM) and the systems containing Pt(iv) complexes, indicating that these 2D cell models might not be reductive enough to completely convert the prodrugs into active drugs. As such, cell viability studies in 3D cell models of the same A549 cell line were performed. 3D models can overcome some shortcomings of the 2D cancer cell cultures and better recapitulate the *in vivo* acidic and reductive tumour microenvironment.^4,64–67^ Results showed no significant difference in the cell viability following treatment with cisplatin, the Pt(iv) prodrug and Fe_3_O_4_@PAA–Pt(iv) nanoparticles (100 μM of Pt, Fig. 6(C)), which confirms that these 3D cell systems better mimic the reductive *in vivo* tumour microenvironments. Altogether, the 2D and 3D cell viability results confirm that the Fe_3_O_4_@PAA–Pt(iv) nanoparticles work as redox responsive therapeutic agents.

A hemolysis assay was performed to assess the blood compatibility of the nanoparticles as a preliminary evaluation for future *in vivo* studies (ESI,† Fig. S6). Across all tested concentrations, hemolysis did not exceed 5%, the maximum level defined by ISO 10993-4 for blood-contacting medical devices. These results suggest good hemocompatibility and support the potential of these nanoparticles as a theranostic probes.

The tested concentrations did not cause hemolysis exceeding 5% – the maximum level allowed by ISO 10993-4 standards for blood-contacting medical devices. This is a promising indicator for further work with these nanoparticles aimed at *in vivo* evaluation of the theranostic probe.

## Conclusions

In this work, Fe_3_O_4_@PAA–Pt(iv) nanoparticles were synthesised as MR theranostics for cancer therapy with environmentally- (redox- and TME-) activated therapeutic capabilities.

Synthesis of the highly-functionalisable Fe_3_O_4_@PAA nanoparticles was achieved using a hydrothermal protocol and yielded nanoparticles with optimal magnetic properties for MR *T*_2_ contrast applications. The PAA coating on the nanoparticles allows for versatile surface functionalisation of the nanoparticles, while also ensuring the stability and biocompatibility of these systems. Thorough characterisation of the Fe_3_O_4_@PAA nanoparticles and preliminary functionalisation studies with fluorescein cadaverine provided information on the concentration of nanoparticles in solution and allowed for the estimation of the number of functionalisable carboxylic acids on the surface of the systems. These pre-studies proved useful when preparing the final Fe_3_O_4_@PAA–Pt(iv) nanoparticles, since controlling the Pt/Fe ratio in these nanoparticles was particularly important to ensure they displayed optimal imaging and therapeutic properties. Optimisation of the synthesis of Fe_3_O_4_@PAA–Pt(iv) nanoparticles allowed for the production of nanoparticles with appropriate hydrodynamic diameter (72 ± 8 nm) and Pt/Fe ratio (0.1) within the optimal range (0.05 to 0.5).

After the characterisation of the Fe_3_O_4_@PAA–Pt(iv) nanoparticles, *in vitro* MR studies confirmed that these nanoparticles were internalised by 2D and 3D models of A549 cells after 6 h of incubation, inducing a strong *T*_2_ contrast. Cell viability studies confirmed that Pt(iv)-functionalised nanostructures could induce apoptosis in cancer cell lines, presenting an IC_50_ of 156.0 μM in 2D cell systems. Moreover, Fe_3_O_4_@PAA–Pt(iv) nanoparticles were proved to be at least as efficient at inducing cell death as cisplatin in more complex and more reductive 3D cell models.

These results present Fe_3_O_4_@PAA–Pt(iv) nanoparticles as smart cancer theranostics for *T*_2_ MR imaging with redox- and TME-activated therapy with potential for further functionalisation.

## Materials and methods

### General

Cisplatin was purchased from TCI Chemicals (Zwijndrecht, Belgium). Dulbecco's Modified Eagle Medium/Nutrient Mixture F-12 (DMEM-F12) was purchased from Gibcol Solutions Ltd (Auckland, New Zealand). Penicillin–streptomycin solution was purchased from Biotecnomica Unipessoal Lda (São Mamede Infesta, Portugal). Fetal bovine serum and trypsin were purchased from ThermoFisher (Massachusetts, USA). All other chemicals and reagents were purchased from Sigma Aldrich (Dorset, UK), and all solvents were purchased from VWR (Leicestershire, UK). All purchased products were used as supplied without any further purification. Water and H_2_O refer to high purity water with resistivity value of 18 MΩ.

Hydrodynamic size and surface charge studies were performed on a Horiba nanoPartica SZ-100 instrument. A JEOL 2010 transmission electron microscope (JEM-2100-HT) working at 200 keV was used to image the nanoparticles. The TEM samples were prepared by depositing nanoparticle aqueous solutions (7 mL) onto 400 mesh carbon coated copper TEM grids (EM Resolutions Ltd, UK) and dried at room temperature for 24 h before use. UV/Vis spectra were recorded using a Shimadzu UV-2550 UV/Vis spectrophotometer. FTIR spectra were recorded using a VERTEX 80v vacuum FTIR spectrometer. XPS measurements were performed on an ESCALAB™ QXi X-ray Photoelectron Spectrometer. The XPS samples were prepared by drop casting onto clean silicon wafers. A spectrometer ICPE-9000 was used to measure the concentration of Fe and Pt. Elemental analysis was performed by the University of Hull elemental analysis service. Powder XRD samples were measured with X-ray diffractometer PANalytical's X’Pert PRO MRD. TGA samples were analysed using Thermogravimetric Analyzer TGA/DSC1/1100 SF. A superconducting quantum interference device magnetometer (SQUID, Quantum Design) was used to study the magnetic properties of iron-based nanoparticles through magnetic field (hysteresis loops)- and temperature (zero-field-cooled and field-cooled)-dependent magnetization measurements.

### Synthesis of PAA-coated iron oxide nanoparticles

Ammonium hydroxide (12 mL, 28–30% in H_2_O) was added to a solution of FeCl_2_·4H_2_O (7.8 mmol) and FeCl_3_·6H_2_O (13.7 mmol) in water (20 mL). Sodium polyacrylic acid (PAANa, 5100 g mol^−1^, 0.39 mmol) was added and the reaction mixture was placed inside a poly(tetrafluoroethylene) (PTFE) vessel and heated to 150 °C for 24 h inside in a stainless-steel autoclave. After cooling down, acetone was added, and the mixture was centrifuged at 4000 rpm for 5 min. The supernatant was discarded, and the pellet re-suspended in water. This process was repeated twice. The final solution was then centrifuged for 3 min at 3000 rpm to remove large aggregates. The supernatant was kept and stored until further use. Nanoparticles were characterised by: TEM: spherical shape, 8.2 ± 1.4 nm diameter, DLS: *D*_H_ = 30.6 ± 3.7 nm, zeta: *ζ*-pot = −86.7 ± 0.9 mV, XRD: 2*θ* (°): 30.064, 35.289, 42.928, 53.530, 57.014, 62.239, FTIR: (cm^−1^) 3364 (*ν*_OH_), 2942 (*ν*_C–H_), 1662 (*ν*_C=O_), 1554 (*ν*_COO_), 1458 (*δ*_C–O–H_), 1396 (*δ*_CH_2__), 1322 (*ν*_C–O_), 864 (*δ*_O–H_), 572, TGA: *n*_organic_/*n*_inorganic_ = 24.6, UV-Vis: (nm) 350–400, and relaxometry (1.5 T): *r*_2_ = 141.60 mM^−1^ s^−1^.

### Synthesis of Pt(iv) precursor

Pt(iv) prodrug dihydroxycisplatin (*cis*,*cis*,*trans*-diamminedichlorodihydroxyplatinum(iv), DHC) was prepared according to previously published methodologies. Briefly, hydrogen peroxide (H_2_O_2_, 30% in water, 70 equiv.) was added dropwise to a bright yellow suspension of cisplatin (1.80 mmol, 1 equiv.) in water inside a microwave vessel. The reaction mixture was heated to 70 °C for 15 min in a CEM Discover SP microwave. The reaction mixture was cooled down and the solvent was removed *in vacuo*. The residue was sequentially suspended in ethanol and diethyl ether to afford a light-yellow powder. Recrystallisation from water provided dihydroxycisplatin as bright yellow crystals (1.11 mmol, 61%). Product was characterised by XRD (ESI,† Fig. S2), FTIR (cm^−1^): 3520 (*ν*_O–H_), 1040 (*δ*_PtO–H_) and 552 (*ν*_PtO_), and EA: calculated (%) for Cl_2_H_8_O_2_N_2_Pt: C 0.00, H 2.41, N 8.39; found (%): C 0.00, H 2.44, N 8.52.

### Determination of molar concentration and molecular weight of nanoparticles

The average inorganic core diameter of the nanoparticles, which was determined by TEM, was used to calculate the volume of a single nanoparticle. Since XRD studies confirmed that the NPs’ core was made of magnetite, the mass of a single particle was estimated by multiplication of the volume of the nanoparticle core and the known density of magnetite (5.17 g cm^−3^). The amount of magnetite in each particle ((Fe_3_O_4_)_*x*_) was calculated by dividing the molecular weight of the NPs’ inorganic core (mass of a single core multiplied by Avogadro's constant) by the molecular weight of magnetite. Using the ICP measurements, the concentration of nanoparticles in solution was calculated, by dividing the molar concentration of Fe in solution by 3 times the mass of a single core. From the TGA experiment, it was possible to determine the ratio of *m*_organic_ to *m*_inorganic_, which was used to estimate the mass of organic component in a single particle and subsequently, the molecular weight of the Fe_3_O_4_@PAA nanoparticles ((*m*_organic_ + *m*_inorganic_)/Avogadro's constant).

### Fluorescein studies

The absorbance of different concentrations of fluorescein cadaverine (0–62.4 μM) was recorded. The absorbance intensity at 492 nm *versus* [fluorescein cadaverine] was plotted, according to the Beer–Lambert law equation, to determine the molar extinction coefficient (*ε*) of fluorescein cadaverine:*A* = *ε* × [Fluorescein cadaverine]

EDC (10 mg, 0.05 mmol) and NHS (6 mg, 0.05 mmol) were added to a solution of Fe_3_O_4_@PAA nanoparticles (1–50 μL) in water (0.1–5 mL), and the reaction mixture was stirred for 30 min. An aqueous solution of fluorescein cadaverine (500 μL, 2 mM) was added and the reaction mixture was stirred for 2 days. The mixture was then centrifuged at 10 000 rpm for 10 min and the supernatant was collected and analysed by UV-Vis. The concentration of unreacted fluorescein cadaverine was determined using the Beer–Lambert equation. The number of moles of fluorescein that reacted with the carboxylic acids of the iron-based nanoparticles was then calculated by subtracting the number of moles of fluorescein left in the supernatant from the number of moles of fluorescein added, after accounting for the dilutions made. The number of moles of COOH/fluorescein cadaverine that reacted in each condition was plotted against the number moles of nanoparticles that were added in each condition, using a non-linear fit.

### Synthesis of Pt(iv)-functionalised PAA-coated iron oxide nanoparticles

EDC (690 mg, 3.6 mmol, 1.2 equiv.) and *N*-hydroxysulfosuccinimide (sulfo-NHS, 730 mg, 3.4 mmol, 1.1 equiv.) were added to a diluted solution of Fe_3_O_4_@PAA nanoparticles (2 mL) in water (98 mL) and the reaction mixture was stirred for 30 min. Dihydroxycisplatin (DHC, 1.02 g, 3.05 mmol) was added and the reaction mixture was stirred for 2 days. Acetone (1 : 1 to 2 : 1 of total reaction volume) was then added and the mixture was centrifuged at 4000 rpm for 5 min. The supernatant was discarded, and the pellet re-suspended in water. This process was repeated twice. The final solution was then centrifuged for 3 min at 3000 rpm to remove large aggregates. The supernatant was kept and stored until further use. Nanoparticles were characterised by: DLS: *D*_H_ = 72.4 ± 8.1 nm, zeta: *ζ*-pot = −1.0 mV ± 0.2 mV, FTIR: (cm^−1^) 3390 (*ν*_NH_3__), 2926 (*ν*_C–H_), 1683 (*ν*_C=O_), 1580 (*δ*_NH_3__), 1455 (*δ*_C–O–H_), 1405 (*δ*_CH_2__), 552 (*ν*_PtO_), UV-Vis: (nm) 260, 350–400 (broad), and relaxometry (1.5 T): *r*_2_ = 215.60 mM^−1^ s^−1^.

### Relaxivity measurements

*T*_2_ relaxation times were measured with a Minispec mq60 relaxometer, at 1.5 T. At least three concentrations were measured for each sample and all experiments were performed at 37 °C and pH = 7.4. Carr–Purcell–Meiboom–Gil (CPMG) sequences were used to measure the transversal relaxation time (*T*_2_). The transversal relaxivity value (*r*_2_, in mM^−1^ s^−1^) was calculated as the slope of the curve fitting 1/*T*_2_ (in s^−1^) *vs.* Fe concentration (in mM).

### Magnetic resonance imaging

MR imaging was performed in a 3.0 T horizontal bore MR Solutions Benchtop MRI system (Guildford, UK) equipped with 48 G cm^−1^ actively shielded gradients. For imaging the sample, a 56 mm diameter quadrature birdcage coil was used in transmit/receive mode. All MR images of the phantoms were acquired with an image matrix 256 × 252, FOV 60 × 60 mm, 3 slices with a slice thickness of 1 mm and with no slice gap. For *T*_2_-weighted imaging, a fast spin echo based (FSE) sequence with the following parameters was used: *T*_E_ = 11 to 70 ms, *T*_R_ = 3000 to 5000 ms, *N*_A_ = 32. Image analysis was performed using ImageJ software.

### Magnetometry measurements

A small sample of dried iron oxide nanoparticles with a known weight (1 to 5 mg) was placed inside gelatine capsules, introduced in standard straw sample holders and attached to the measuring rod. Field-dependent magnetization curves of iron oxide nanoparticles were recorded in a superconducting quantum interference device magnetometer (SQUID, Quantum Design), in a magnetic field ranging from −20 to +20 kOe at 5 and 300 K. Zero-field-cooled and field cooled (ZFC–FC) magnetization curves of nanoparticles were recorded in the same magnetometer over the temperature range 2–300 K and under an applied magnetic field of 100 Oe. The magnetization units were expressed as emu per gram of sample.

### *In vitro* cellular uptake studies

A549 cells were seeded in 6-well plates at a density of 2 × 10^5^ cells per well and incubated for 24 h. The medium was then removed, compounds of interest were added at a concentration of 50 μM of Pt and 500 μM Fe and the cells were incubated for 6 h. The cells were washed thrice with PBS and either: (1) treated with HCl (12 M, 1 mL) overnight and diluted to 10 mL before being analysed by ICP-OES; (2) trypsinised, pelleted, transposed to a 200 μL Eppendorf and imaged on MRI. An FSE sequence with the following parameters was used: *T*_E_ = 11 ms, *T*_R_ = 12 000 ms, *N*_A_ = 32. Image analysis was performed using ImageJ software.

### *In vitro* cellular cytotoxicity studies

For 2D cell viability studies, A549 cells were seeded in 96-well plates at a density of 5000 cells per well in 100 μL of complete DMEM F-12 medium and incubated in a 5% CO_2_ atmosphere at 37 °C for 24 h. The culture medium was then removed and replaced with 100 μL of medium containing compounds of interest at different concentrations. The cells were incubated for 48 h. Resazurin was added to each well and the cells were incubated for another 4 h to allow viable cells to reduce the non-fluorescent blue resazurin to red fluorescent dye resorufin. The fluorescence was measured at 590 nm by using a Biotek Synergy H1 Microtiter Plate Reader (*λ*_ex_ = 560 nm, *λ*_em_ = 590 nm).

For 3D cell viability studies, A549 cells were seeded in BIOFLOAT™ 96-well plates (purchased from faCellitate) at 10 000 cells per well in 200 μL of complete DMEM F-12 medium. The plates were centrifuged at 1200 rpm for 5 min and then incubated for 3 days; medium was changed as needed. The spheroids were photographed using a phase-contrast microscope. The culture medium was then replaced with 200 μL of medium containing the compounds of interest at different concentrations. The cells were incubated for 48 h and were then photographed. Resazurin was added to each well and the cells were incubated for another 12 h. The fluorescence was measured at 590 nm by using a Biotek Synergy H1 Microtiter Plate Reader (*λ*_ex_ = 560 nm, *λ*_em_ = 590 nm).

### Hemolysis assay

500 μL of whole blood were mixed with 500 μL of either 10 mM PBS pH 7.4 solution containing serial dilutions of the nanoparticles (0 to 750 mM Fe) or water as positive hemolytic control. The samples were incubated at 37 °C for 90 min before they were centrifuged (600*g*, 5 min). The pellet was discarded and the supernatant incubated in air at room T to promote hemoglobin oxidation. After this period the samples were centrifuged once more (13 400 rpm, 5 min) to remove interfering NPs, and the absorbance of the supernatants was recorded at 560 nm.

## Author contributions

Dr Brito conducted the studies, performed formal analysis of the results, and wrote the original draft. Cátia Rocha performed some of the studies and experiments. Dr Price, Dr Bañobre-López, Dr Gallo and Dr Stasiuk contributed to methodology development and result interpretation. Dr Stasiuk, Dr Gallo and Dr Bañobre-López conceptualized the study, secured funding, and supervised the project. All authors reviewed, edited and approved the final manuscript.

## Conflicts of interest

There are no conflicts to declare.

## Supplementary Material

TB-013-D5TB01007A-s001

## Data Availability

The data supporting this article have been included as part of the ESI.†
