# Supplementary material for: Pt(iv)-functionalised polyacrylic acid-coated iron oxide magnetic nanoparticles as redox-responsive cancer theranostics
Source: J Mater Chem B. 2025 Jul 1;13(30):9217–27. doi: 10.1039/d5tb01007a (PMC12227113; doi:10.1039/d5tb01007a)
Supplement: TB-013-D5TB01007A-s001 [file TB-013-D5TB01007A-s001.pdf]

Supplementary Information (SI) for Journal of Materials Chemistry B.
This journal is © The Royal Society of Chemistry 2025

Supplementary Information

Title: Pt(IV)-functionalised polyacrylic acid-coated iron oxide magnetic nanoparticles as redox-responsive cancer theranostics.

Authors: Beatriz Brito,[a,b,c] Thomas W. Price,[a] Cátia Vieira Rocha,[b] Manuel Bañobre-López[b], Graeme J. Stasiuk[a*] and Juan Gallo[b*]

Affiliations: [a]Department of Imaging Chemistry and Biology, School of Biomedical Engineering and Imaging Sciences, King's College London, Strand, WC2R 2LS London, UK graeme.stasiuk@kcl.ac.uk, [b]Advanced Magnetic Theranostic Nanostructures Lab, International Iberian Nanotechnology Laboratory, Av. Mestre José Veiga, 4715-330 Braga, juan.gallo@inl.int, [c]School of Life Sciences, Faculty of Health Sciences, University of Hull, Cottingham Road, HU6 7RX Hull, UK

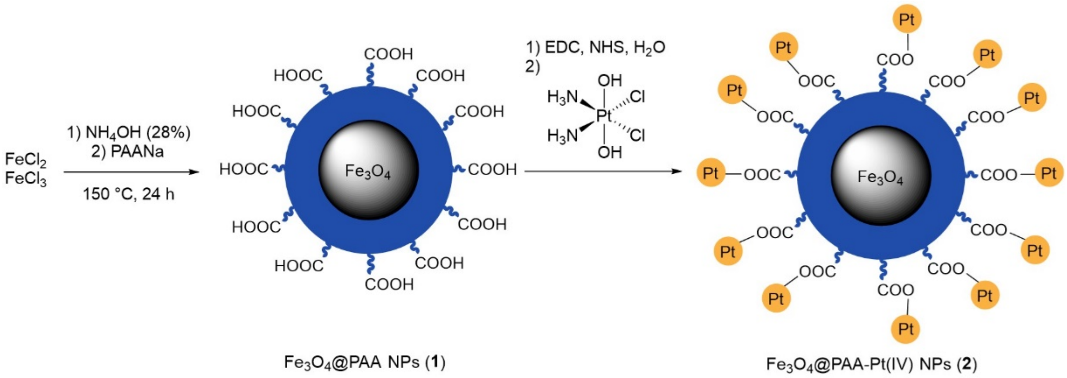

Scheme S1. Synthesis of Fe_3O_4@PAA NPs and Fe_3O_4@PAA-Pt(IV) NPs

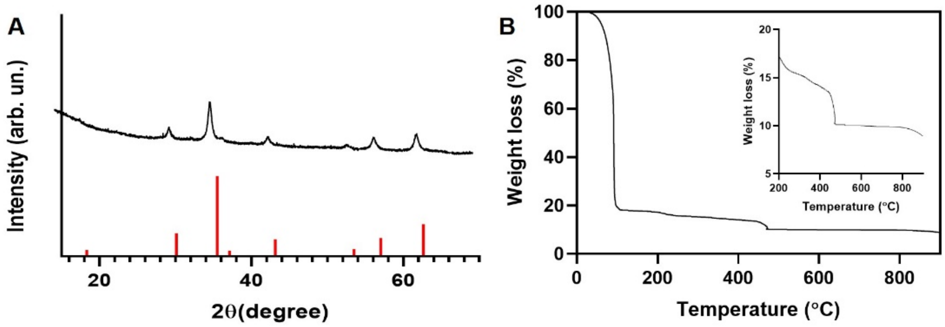

C

D_H (nm)	Core size (nm)	[Fe] (mM)	$m_{organic}/m_{inorganic}$	MW_{calc} (g/mol)
31 ± 4	8 ± 1	284.7	0.84	1,686,879.96

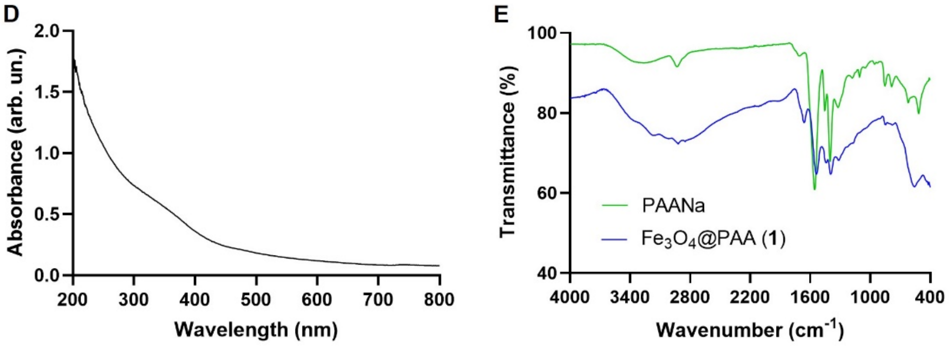

Figure S1. A) Powder XRD patterns of the synthesized NPs (black) and patterns corresponding to the positions of the Bragg reflections expected for magnetite (red). **B)** Thermogravimetric analysis (TGA) of Fe_3O_4@PAA nanoparticles and insert of zoomed-in region from 200 to 800 °C. **C)** Summary table of DLS, TEM, ICP and TGA results. **D)** UV-vis spectrum for **18** in H_2O (pH = 7.4). **E)** FTIR spectra of nanoparticles **18** and PAANa precursor.

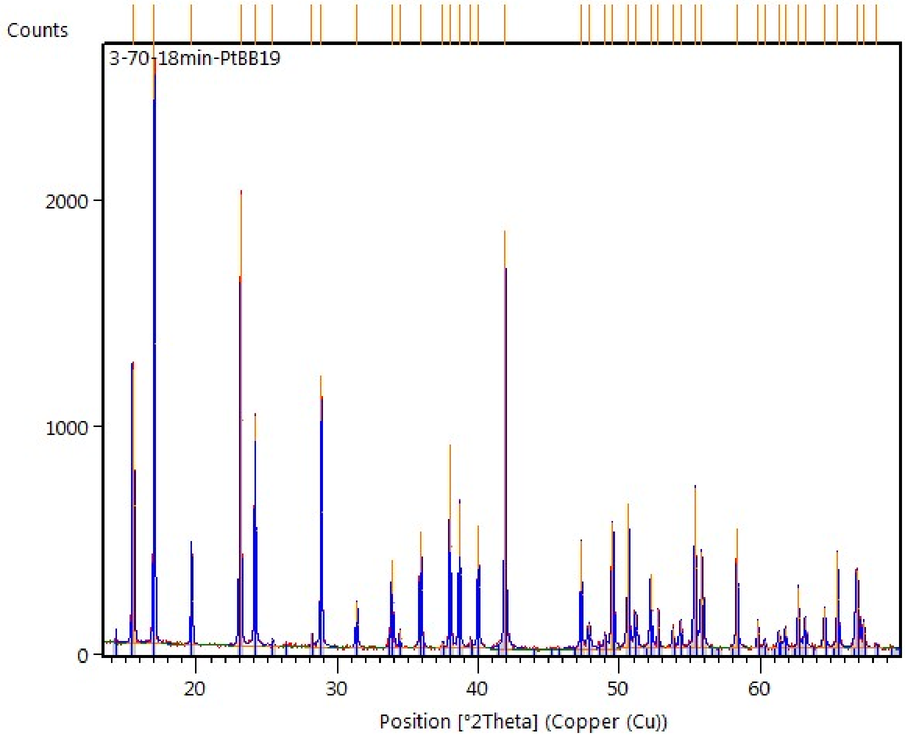

Figure S2. XRD spectrum of Pt(IV) prodrug oxoplatin (cis, cis, trans-Diamminedichlorodihydroxyplatinum(IV)).

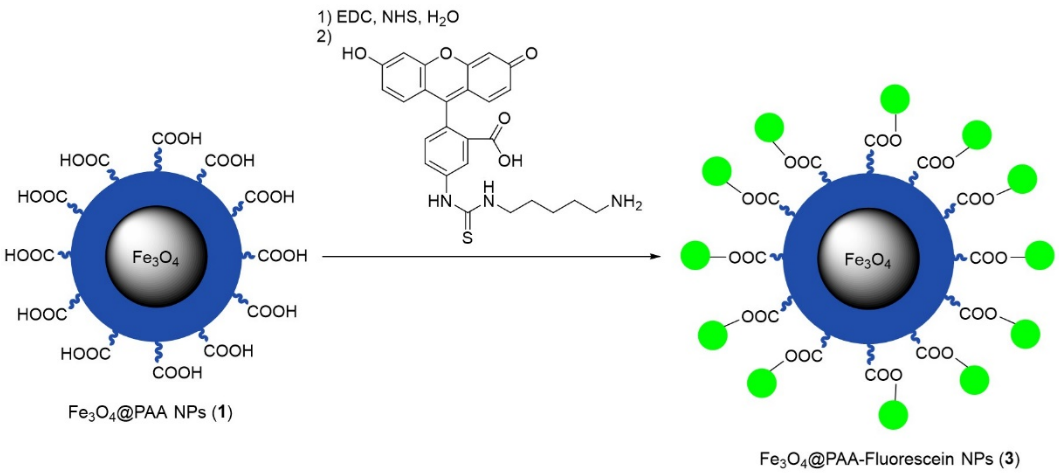

Scheme 2. Synthesis of Fe_3O_4@PAA-Fluorescein nanoparticles

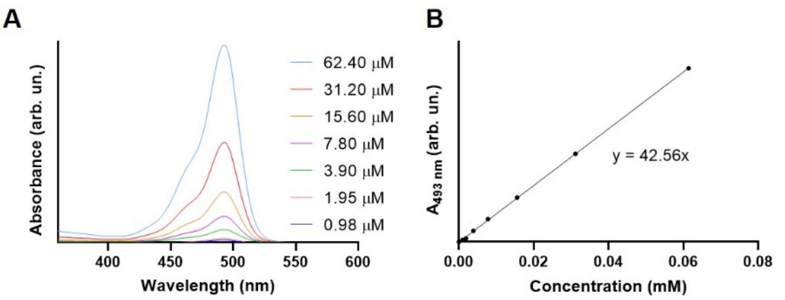

D

V_{NPs} (µL)	$A_{492\ nm}$ (arb. un.)	[Fluorescein] (mM)	$n_{leftover\ Fluorescein}$ (mmol)	$n_{COOH\ reacted}$ (mmol)
1	2.15	5.05×10^{-2}	9.81×10^{-4}	-1.11×10^{-5}
2	1.90	4.46×10^{-2}	8.66×10^{-4}	1.04×10^{-4}
5	1.55	3.64×10^{-2}	6.92×10^{-4}	2.28×10^{-4}
10	1.31	3.09×10^{-2}	7.42×10^{-4}	2.58×10^{-4}
25	0.66	1.56×10^{-2}	3.74×10^{-4}	6.26×10^{-4}
50	0.32	7.55×10^{-3}	1.81×10^{-4}	8.19×10^{-4}

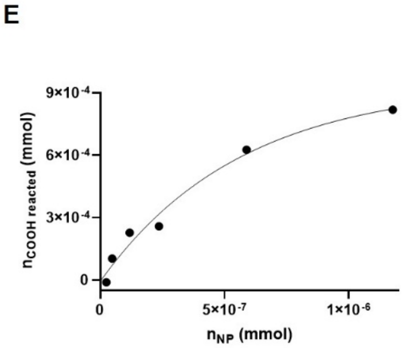

Figure S3. A) UV-vis spectra of standard solutions of fluorescein cadaverine in H_2O. **B)** Linear regression relating absorbance at 493 nm versus concentration of fluorescein cadaverin, $R^2 = 0.9998$. **C)** UV-vis spectra of supernatants from different reactions containing varying amounts of nanoparticles **1**, in H_2O. **D)** Table showing the measured absorbance values for each reaction condition and corresponding concentration and number moles of fluorescein in each reaction mixture, as well as calculated number carboxylic acids (COOH) in nanoparticles **3** that have reacted. **E)** Non-linear regression relating the number of moles of COOH reacted with the number of moles of nanoparticles in solution, $R^2 = 0.9836$.

Table S1. Effect of Pt(IV) complex in hydrodynamic size (D_H) of Fe_3O_4@PAA-Pt(IV) nanoparticles.

Reaction	V_{NPs} (µL)	$n_{EDC\ and\ NHS}$ (mmol)	n_{DHC} (mmol)	D_H (nm)
A	10	6.8×10^{-2}	4.5×10^{-2}	649.8 ± 89.7
B			3.0×10^{-2}	510.2 ± 69.0
C			3.0×10^{-3}	73.3 ± 9.1
D			1.5×10^{-3}	89.6 ± 16.6

Table S2. Optimisation of nanoparticle concentration in reaction mixture for the synthesis of Fe_3O_4@PAA-Pt(IV) nanoparticles.

Reaction	V_{NPs} (µL)	n_{NPs} (mmol)	$n_{EDC\ and\ NHS}$ (mmol)	n_{DHC} (mmol)	$DHC/NP_{reaction}$	$V_{Reaction}$ (mL)	D_H (nm)	Pt/Fe_{ICP}	Pt/NP_{ICP}
A	100	2.4×10^{-6}	1.0×10^{-2}	7.0×10^{-4}	291	10	55 ± 10	0.008	95
B				7.0×10^{-3}	2914	10	56 ± 9	0.03	356
C					2914	1	766 ± 56	0.04	474

Table S3. Optimisation of Pt/Fe ratio in the synthesis of Fe_3O_4@PAA-Pt(IV) nanoparticles.

Reaction	V_{NPs} (μL)	n_{NPs} (mmol)	$n_{EDC\ and\ NHS}$ (mmol)	n_{DHC} (mmol)	$DHC/NP_{reaction}$	$V_{Reaction}$ (mL)	Pt/Fe_{ICP}	Pt/NP_{ICP}
A	100	1.4×10^{-5}			413	10	0.02	40
B	75	1.1×10^{-5}			545	7.5	0.02	46
C	50	7.2×10^{-6}			833	5	0.04	81
D	25	3.6×10^{-6}	6.0×10^{-3}	3.0×10^{-3}	1 666	2.5	0.08	154
E	10	1.4×10^{-6}			2 285	1	0.06	123
F	5	7.2×10^{-7}			8 333	0.5	0.2	453
G	2	2.9×10^{-7}			20 689	0.2	0.4	801

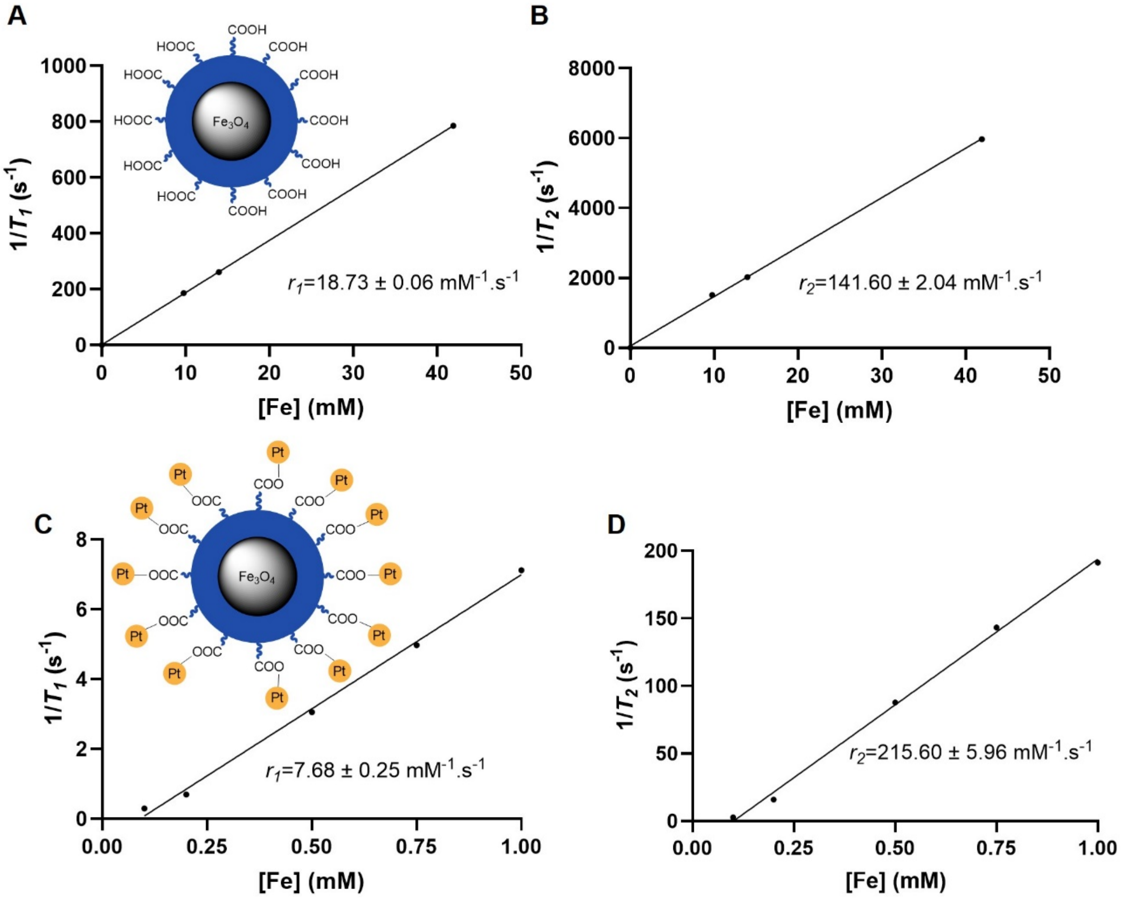

Figure S4. Linear regression fitting data from **A)** T_1 measurements for Fe_3O_4@PAA NPs, **B)** T_2 measurements for Fe_3O_4@PAA NPs, **C)** T_1 measurements for Fe_3O_4@PAA-Pt(IV) NPs, **D)** T_2 measurements for Fe_3O_4@PAA-Pt(IV) NPs, at 25 °C, in H_2O, pH = 7.4 and corresponding r_1 and r_2 values, at 1.5 T (R^2 = 1.0000, 0.9996, 0.9969 and 0.9977 respectively).

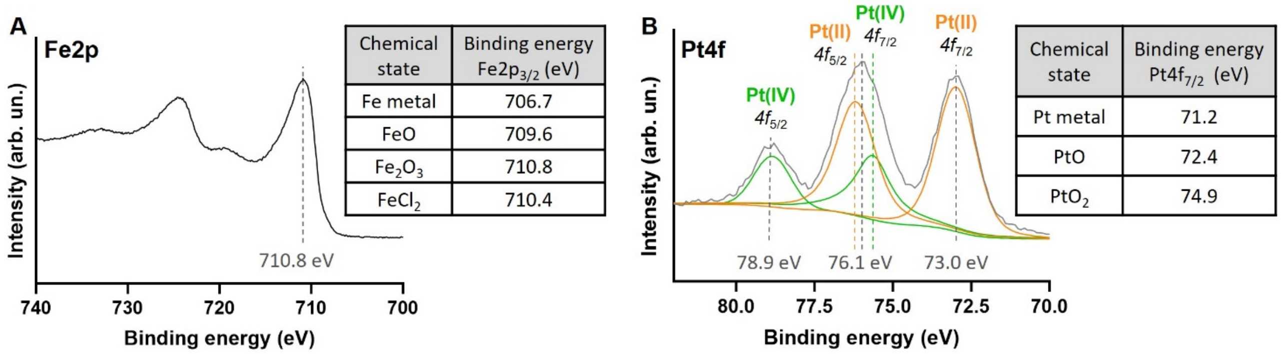

Figure S5. A) Expanded Fe2p region of XPS spectra of Fe_3O_4@PAA-Pt(IV) nanoparticles and table with reference binding energies of common Fe states. **B)** Expanded Pt4f region of XPS spectra of NPs **2** and table with reference binding energies of common Pt states.

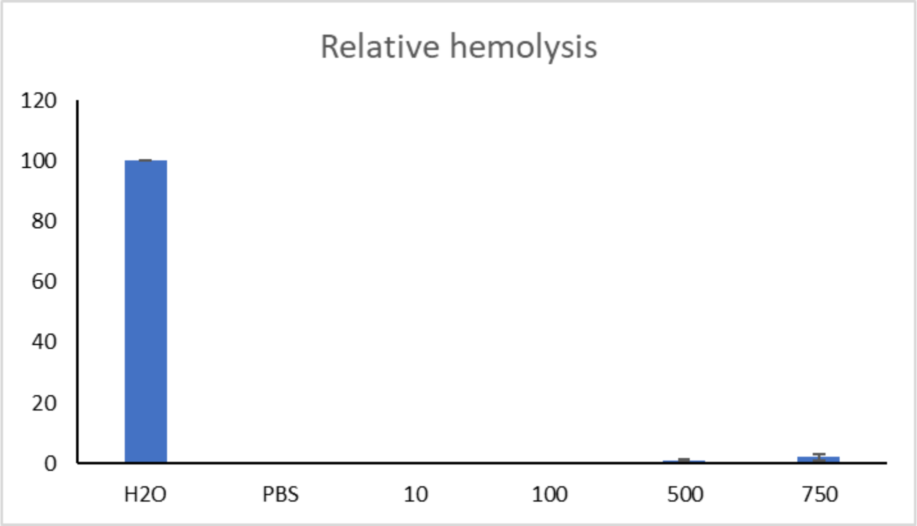

Figure S6. Hemolysis assay with whole blood. Concentration of Fe in nanoparticles from 0 to 750 mM.
